# Automatic Fabric Defect Detection Using Cascaded Mixed Feature Pyramid with Guided Localization

**DOI:** 10.3390/s20030871

**Published:** 2020-02-06

**Authors:** You Wu, Xiaodong Zhang, Fengzhou Fang

**Affiliations:** State Key Laboratory of Precision Measuring Technology & Instruments, Centre of Micro/Nano Manufacturing Technology—MNMT, Tianjin University, Tianjin 300072, China

**Keywords:** fabric defect, object detection, mixed kernels, cross-scale, cascaded center-ness, deformable localization

## Abstract

Generic object detection algorithms for natural images have been proven to have excellent performance. In this paper, fabric defect detection on optical image datasets is systematically studied. In contrast to generic datasets, defect images are multi-scale, noise-filled, and blurred. Back-light intensity would also be sensitive for visual perception. Large-scale fabric defect datasets are collected, selected, and employed to fulfill the requirements of detection in industrial practice in order to address these imbalanced issues. An improved two-stage defect detector is constructed for achieving better generalization. Stacked feature pyramid networks are set up to aggregate cross-scale defect patterns on interpolating mixed depth-wise block in stage one. By sharing feature maps, center-ness and shape branches merges cascaded modules with deformable convolution to filter and refine the proposed guided anchors. After balanced sampling, the proposals are down-sampled by position-sensitive pooling for region of interest, in order to characterize interactions among fabric defect images in stage two. The experiments show that the end-to-end architecture improves the occluded defect performance of region-based object detectors as compared with the current detectors.

## 1. Introduction

Industrial defect detection is important in manufacturing. Specifically, fabric defect control is the main content of quality control in the textile industry, which would significantly increase the additional processing costs of the fabric. The cost is derived from manual positioning and the detection of defects and suspending to remove them. On the one hand, manual quality inspections are inefficient and they must often be seen under good backlighting. On the other hand, there is no quantitative defect classification indicator or boundary. This can result in false or mis-detection, and it is not conducive to the late repair of defects or the removal of defects before they occur.

With the popularization of artificial intelligence, the automatic detection algorithm is gradually replaced by the data-based intelligent learning algorithm from the traditional extraction method based on feature values and low-dimensional pixel features. When compared with the traditional algorithm, the heuristic learning algorithm has the advantages of high recognition precision, strong generalization ability, no need to construct complex analytical relations, and small sensitivity range for hyper-parameters. The intelligent detecting methods are divided into unsupervised learning and supervised learning, both of which have gained good performance in defect detection. For the former, Ahmed et al. [1] proposed to conduct the low rank and sparse decomposition jointly and extract weaker defects feature based on wavelet integrated alternating dictionary matrix transformation; Gao et al. [2] utilized an unsupervised sparse component extraction algorithm to detect micro defects in a thermography imaging system by building an internal sub-sparse grouping mechanism and adaptive fine-tuning strategy; Wang et al. [3] established a successive optical flow for projecting the thermal diffusion and constructed principal component analysis to further mine the spatial-transient patterns for strengthening the detectability and sensitivity; Hamdi et al. [4] utilized non-extensive standard deviation filtering and K-means to cluster fabric defect block; Mei et al. [5] reconstruct fabric defect image patches with a convolutional denoising auto-encoder network at multiple Gaussian pyramid levels and to synthesize the detection results from the corresponding resolution channels. For the latter, detecting methods that are mainly based on convolutional deep learning [6] and researchers take an effort in optimizing architecture. Li et al. introduced DetNet [7], which was specifically designed to keep high resolution feature maps for prediction with dilated convolutions to increase receptive fields. Ren et al. proposed the Region Proposal Network (RPN) [8] to generate proposals in a supervised way based on sliding convolution filters. For each position, anchors (or initial estimates of bounding boxes) of varying size and aspect ratios were proposed. Liu et al. [9] learned a lightweight scale aware network to resize images, such that all objects were in a similar scale. Singh et al. [10] conducted comprehensive experiments on small and blurred object detection. Girshick et al. [11] proposed the Region of Interest Pooling (RoI-Pooling) layer to encode region features, which is similar to max-pooling, but across (potentially) different sized regions. He et al. [12] proposed RoI-Align layer, which addressed the quantization issue by bilinear interpolation at fractionally sampled positions within each grid due to the misalignment of the object position from down-sampling operation. Based on RoI-Align, Jiang et al. [13] presented PrRoI-Pooling, which avoided any quantization of coordinates and it had a continuous gradient on the bounding box coordinates.

Although generic models are simple and easy to deploy, they are either over-fitting or feature extracting insufficiency. More targeted detectors for defect images need to be re-established, owing to the dependence on the quality and manifold distribution of the dataset. A variety of imbalances in the image data structure lead to the difficulty of defect identification. The main contributions of this work lie in several aspects due to the misalignment of the object position from down-sampling operation. Firstly, a large imbalanced fabric defect dataset is collected and especially selected for training and validating a robust detector. Secondly, an efficient architecture for defect detection is well-designed, along with improved sub-modules from generic architectures for object detection. Third, the experiments reveal that overfitting and feature extracting sufficiency are the main causes for the accuracy loss of defect detection. By simplifying models, not only is the accuracy improved, but parameter space is also compressed, which reduces the inference delay and lays the foundation of industrial real-time defect detection.

The remainder of this paper is structured, as follows: Section 2 introduces a dataset well-designed on principles of imbalanced variance of real-scene fabric defect images aiming at exploring a robust detector framework and validating its generalization when compared with others. Section 3 reveals the general network configuration for fabric defect detection. A simplified backbone with mixed convolution is proposed for avoiding over-fitting, a composite interpolating pyramid is used for deep feature fusion and a cascaded center-ness refining block is provided for localization regression. Section 4 states the experimental settings and related work in the comparison between proposed network and other generic ones. The proposed modules for fabric defect are estimated effective on location by an ablation study. Finally, Section 5 concludes the paper.

## 2. Data Space

The feature unwrapping of high-dimensional abstract data space is the core task of deep learning. The data set connects the real scene and semantic algorithm. Therefore, the sample space approximation considers the real space, according to the maximum likelihood principle. This study trains the generalization ability in the corresponding unbalance of real data by constructing the imbalance of sample data. To date, there is still no fabric defect dataset that is adequate and classic in the size, class, and background variations. This paper proposes large-scale optical fabric images, which is named as Fabric Defects (FBDF) and consists of 2575 images covered by 20 defect categories and 4193 instances, in order to address the aforementioned problems. All the raw defect instances and fabric images are collected from textile mills in Guangdong Province, China. These images are selected from hundreds of fabric products with classical defects and labeled in 20 classes according to product demand and expert experience. Details and access are available in https://github.com/WuYou950731/Fabric-Defect-Detection.

### 2.1. Defect Class Selection

Selecting appropriate texture defect classes is the first step of constructing the dataset. Ambiguous category and bounding is one of the major issues for industrial datasets, in other words, they are too blurred to label accurately, despite experts making sure that it is a defect. Therefore, defect classes selected need to be high-resolution and relatively salient when compared with background and other categories. Some categories that are not common in real-world applications are not included in FBDF and some fine-grained categories are considered as a child category. For example, some stains that are common, clear, and play an important role in textile manufacturing environment analysis, such as oil stains, rust strains, and dye stains, are labeled as the same. In addition, most of the defect categories in existing datasets are selected from gray cloth, which are from the substrate with primary colors rather than decorative patterns. However, defects appear not only in the production stage, but also in transportation, sorting, and even cutting. Therefore, different patterns and backgrounds would be taken into account in FBDF beside texture of fabric, which could be the interference of detectors. By overall selecting classes and image properties, Table 1 shows the samples of FBDF.

### 2.2. Characteristics of FBDF Dataset

A good detector should be of high generalization ability and a good dataset should be a benchmark and guidance in testing and training, respectively. When dataset is relatively interior balanced [14], especially for natural images, such as MS-COCO (Microsoft Common Objects in Context) [15] and Pascal-VOC (Pattern Analysis, Statistical Modelling and Computational Learning Visual Object Classes) [16], different architectures of detectors published have similar performance. However, for industrial dataset, which is seriously imbalanced in image quality, size, and background, some techniques do not work anymore. It is necessary to introduce the imbalance into FBDF in order to train the recognition capability of defect detector. Four key characteristics are highlighted.

**Figure 1:**
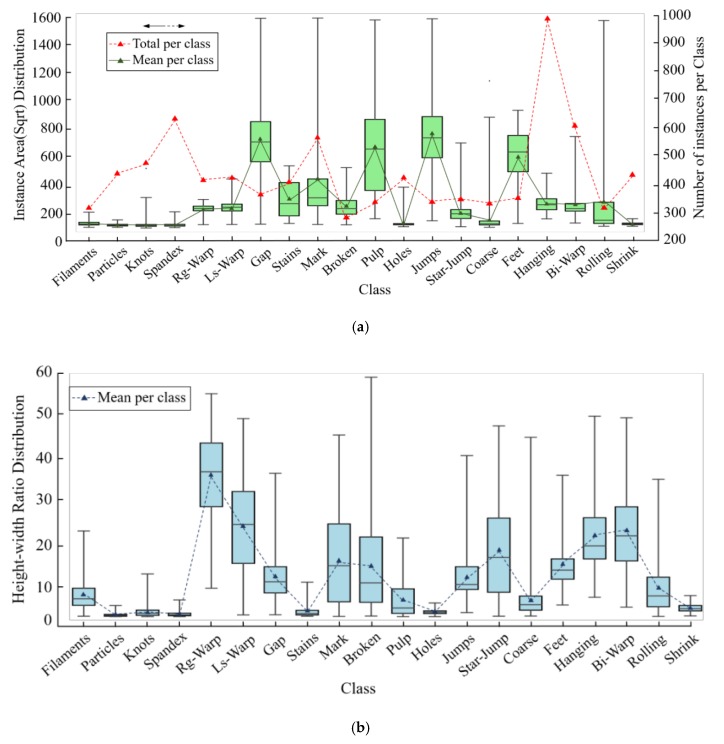
Imbalance distribution of instances for FBDF. (**a**) Area and number distribution of instances per class. (**b**) Size ratio distribution of bounding boxes per class.

- **Large scale**. FBDF consists of 2k optical fabric images and 4k defect instances that are manually labeled with axis-aligned bounding boxes. The size of images is all 2446 × 1000 pixels and the spatial resolution could be down to 0.5 mm. FBDF is collected from the Ali Cloud by the experts in the domain of textile engineering.
- **Instance size and number variations**. Spatial size variation represents actual feature of fabric defects in industrial scene. This is not only because of the spatial resolutions of sensors, but also due to between-class size variation (e.g., “Knots” vs. “Indentation Marks”) and within-class size variation (e.g., “Rough Warp” vs. “Loose Warp”). There is a large range of size variations of defect instances in the proposed FBDF dataset, as shown in Figure 1. For each class of fabric, area, height-width ratio, and the number of instances are various and widely ranged. Few-shot recognition ability of detectors could be validated by number variation; multi-scale recognition ability is from area and height-width ratio variation.

- **Image variations.** A highly desired characteristic for any defect detection system is its robustness to image variations, concerning different textile, cloth pattern, back-light intensity, imaging conditions, etc. Textile is mainly from denim, muslin, satin and so on. Back-light is controlled to guarantee the sharpness of images. Because of the variations in viewpoint, translation is not that important as compared to illumination, background, and defect appearance for each defect class, so they are simplified in FBDF.
- **Inter-class similarity and intra-class diversity.** Inter-class similarity leads to False Positive (FP) and intra-class diversity leads to False Negative (FN) in classifying module of detectors. Comparable defect images in different class are collected without salient modification to obtain the former. To increase the latter, different defect colors, shapes, and scales are taken into account when selecting images. “Spandex” instances present distinguished shapes, and “Jumps” and “Star-jumps” instances are the opposite.

To sum up, FBDF are designed with the purpose of containing imbalanced image data, which are common and practical in textile scenes. FBDF provides a criterion data space for them to learn, fit, and represent to make detectors adapt to various environments, sizes, and classes.

## 3. Methodology

The state-of-the-art object detectors with deep learning can be mainly divided into two major categories: two-stage detectors and one-stage detectors. For a two-stage detector, in the first stage, a sparse set of proposals is generated; and, in the second stage, deep convolutional neural networks encode the feature vectors of generated proposals, followed by making object class predictions. A one-stage detector does not have a separate stage for proposal generation (or learning a proposal generation). They typically consider all positions on the image as potential objects, and they try to classify each region of interest as either background or a target object. Although two-stage detectors generally fall short in terms of lower inference speeds, they often reported state-of-the-art results on dark and low-saliency defect detection.

**Figure 2:**
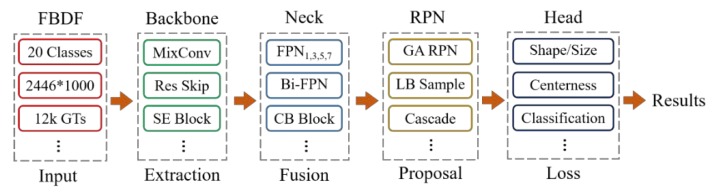
End-to-end fabric defect detection architecture.

As shown in Figure 2, an end-to-end defect detector is composed by data input, backbone for feature extraction, neck for feature fusion and enhancement, RPN for anchor generation, and head for training or inference. In this section, more details of the framework and learning strategies of fabric defect detection application will be introduced.

### 3.1. Backbone for Feature Extraction

Detectors are usually trained based on high-dimension semantic information by adopting convolutional weights to reserve image spatial transformation. R-CNN (Region Convolutional Neural Network) [11] showed the classification ability of backbone is consistent with the location ability in detecting. Moreover, the amount of backbone parameters, which are positive correlation with detection performance, is majority of that in detectors. In this section, the trade-off between latency and accuracy of final forward inference is the main design principal. A lightweight mixed depth-wise convolutional network is introduced for enhancing feature extraction within one single layer, since the imbalance of multi-scale features derived from fabric defect.

Mixed convolution [17] utilized different receptive fields to fuse multi-scale local information by diverse kernel and group sizes. Squeezed and excited [18] branch distinguished the salience of feature layer with visual attention mechanism [19] and residual skip connection [20] deepened semantic extraction and decoding. Inspired by these works, the configuration of network would contain these sub-models and be adjusted for textile dataset in order to lower the burden of RoI extractor to recognize occluded and blurred objects.

**Figure 3:**
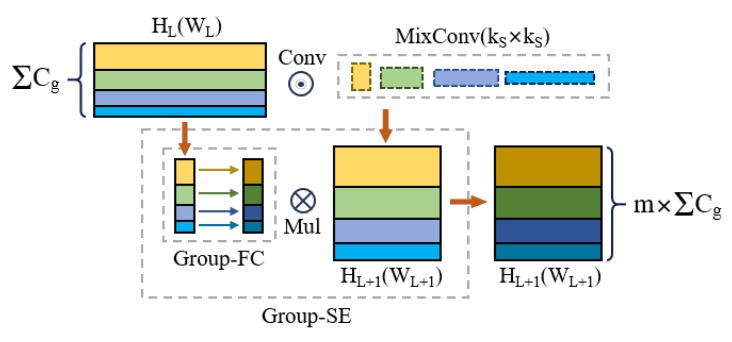
Mixed convolutional module for feature extraction.

Mixed convolution (MC) partitions channels into groups and applies different kernel sizes to each group, as shown in Figure 3. Group size g determines how many different types of kernels to use for a single input tensor. In the extreme case of g = 1, a mixed convolution becomes equivalent to a vanilla depth-wise convolution [21,22,23]. The experiments reveal that g = 5 is generally a safe choice for defect detection, which is size-imbalanced and the maximum area ratio is near 25, as illustrated in Figure 1a. For kernel size per group, if two groups share the same kernel size, it is equivalent to merging these two groups into a single one. Hence, each one should be restricted in different kernel size. Furthermore, kernel size is design to starts from 3 × 3, and monotonically increases by two per group, since small-size kernels generally possess less parameters and floating-point operations per second (FLOPS). Under this circumstance, the kernel size for each group is predefined for any group size g, thus simplifying the designing process. On the other hand, for channel size per group, exponential partition is more generalized than equal partition, since a smaller kernel size fuses less global information, but acquire more channels to compensate local details.

Table 2 states the main specification for feature extraction backbone. SE denotes whether there is a squeezed excited module in that block. AF means the type of nonlinear activation function. Here, HS is for h-swish [24] and RE for ReLU. Batch normalization is used after convolution operations. The stride could be deduced from other information of layer and, here, would be passed over. EXP Size denotes the expansion of the convolution inherit from MobileNetv2 [23], which avoids the loss of pixel feature appeared in ResNet, and the number of elements in the list reveals the times while using Bneck [25]. The FPN column illustrates whether there is a head to introduce the feature map to FPN layers [26]. Operators are mainly mixed convolution parameters and {3 × 3, 5 × 5, 7 × 7} means that the group number is 3 and they are filtered by these kernels, respectively.

### 3.2. Neck for Feature Integrating and Refining

Multi-scale feature fusion aims to aggregate features at different resolution necks. Formally, the multi-scale feature map of conventional FPN is defined as an iteration term:

(1) $$P_{i}^{out}=Conv\left(P_{i}^{in}+Sample\left(P_{i+1}^{out}\right)\right)$$

in which,

(2) $${\vec{P}}^{in}=\left(P_{l_{1}}^{in},P_{l_{2}}^{in},\ldots \right)$$

*Sample* is an up-sampling or down-sampling operation for resolution matching, and *Conv* represents convolutional feature processing. In Equation (1), $p_{i}^{in}$ is the *i*-th input feature layer with different pixel resolution. $p_{i}^{out}$ is the *i*-th output feature layer in the other back propagation path with the same resolution as $p_{i}^{in}$. In Equation (2), ${\vec{P}}^{in}$ represents the parallel feature input flow with interpolating scales. As the foundation of scale fusion, FPN offers two crucial conclusions for defect detection use. Firstly, the defect instance of any scale could be unified to the same resolution as long as stride and kernel are well designed. Secondly, the sampling feature could reserve the most useful information of defect image and defect position is different from background mainly lies in its pixel brightness. This is slightly inconsistent with natural image dataset and more approaching to the principle of maximum pooling. However, the simple top-down FPN is inherently limited by the one-way feature flow.

**Figure 4:**
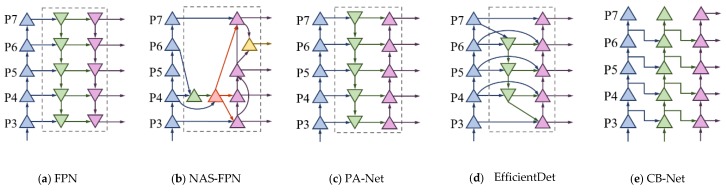
Feature pyramid network frameworks.

Fusing layers need to be cross-scale connected with each other, which derived from compression or interpolation, to continue the strengthening features. Additionally, fusion operation focuses on two aspects, aggregation path, and expansion path. For the former, PANet [27] adds an extraction bottom-up path and CBNet [28] overlays parallel feature maps in different size. The latter outperforms the former for defect classes in detection, since CBNet possesses more parameters and more aggregating feature, as illustrated in Table 3. For the latter, as shown in Figure 4, NAS-FPN [29] treats up-sampling equally to convolution by employing neural architecture search and utilize large-ratio connection to deepen the above two operations. However, simplified configuration, especially unexplained topology of NAS [30] detectors and EfficientDet [31] series, takes the efficiency as the loss of semantic precision.

**Figure 5:**
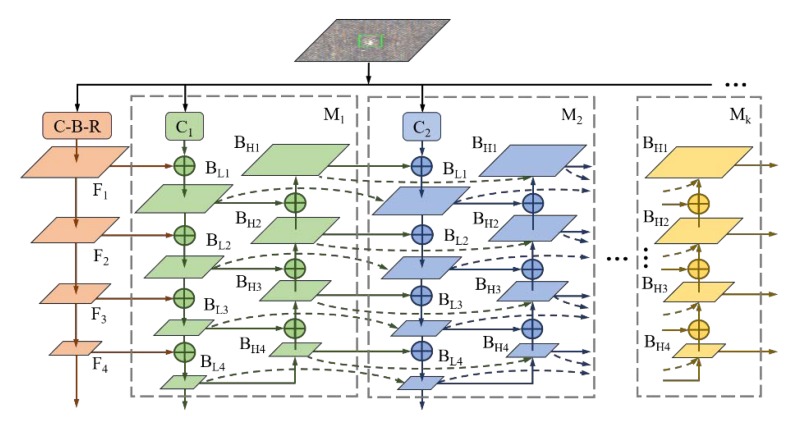
Composite interpolating feature pyramid (CI-FPN).

For low-salience defects, this paper proposes several designing principles for neck feature fusion:

(1) **Compress feature extraction maps.** Defect images share a common dark background and pixel values of object instance have no big difference, so there is no need to enlarge the number of kernels for a layer.
(2) **Add cross-scale fusion without extra computations.** Nodes derived from one input edge are supposed to remove for its low-level semantic representation and aggregation between input and output from the same level made defect region more visually clear.
(3) **Repeat bidirectional (top-down & bottom-up) block.** Unlike PANet, which only has one bidirectional block, the network that is proposed in this paper advices cascaded modules to enable more high-level feature fusion.
(4) **Keep no same lateral size in a repeated block.** Unlike EfficientDet (stacking Bi-FPN) that keep the lateral sizes of up-sampling and down-sampling the same, this paper applies the interpolating layer for approximately continuous extension of scale, as shown in Figure 5. Feature loss could be reduced with least amount of extra latency by this way.

After setting the general fusion operation, weighted pixel analysis is necessary for feature refinement. Since different input layers are at distinguished resolutions, they usually unequally contribute to the output. Previous fusion methods treat all inputs equally without distinction and cause bounding regression drift. Based on conventional resolution resizing and summing up, the paper proposes to weight the salience of feature layer, as follows:

(3) $$B_{L}^{j}(i)=Conv\left(\frac{w_{1}\cdot B_{L}^{j-1}(i)+w_{2}\cdot S_{L}^{j}\left(B_{L}^{j}(i)\right)+w_{3}\cdot S_{H}^{j-1}\left(B_{H}^{j-1}(i-1)\right)}{w_{1}+w_{2}+w_{3}+\epsilon }\right)$$

(4) $$B_{H}^{j}(i)=Conv\left(\frac{w_{1}\cdot S_{L}^{j}\left(B_{L}^{j}(i+1)\right)+w_{2}\cdot S_{H}^{j}\left(B_{H}^{j}(i+1)\right)}{w_{1}+w_{2}+\epsilon }\right)$$

In Equations (3) and (4), the feature flow between *M_i_* and *M_i_*_+1_ mainly build up on two kind of blocks *B_L_* and *B_H_*, which reveal the *j*-th module, *i*-th layer, and extraction path as well as interpolating path. Moreover, learnable weight *w_i_* is scalar in the feature level, which is comparably accurate to tensor in pixel level, yet with minimal time costs. Normalization is resorted to bound the data fluctuation and h-swish replaces Softmax to assign probability to each weight here ranges from 0 to 1 and it alleviates the truncation error at the origin point. ε = 0.001 is a disturbance constant to avoid numerical instability.

### 3.3. Anchor Sampling and Refining

**Figure 6:**
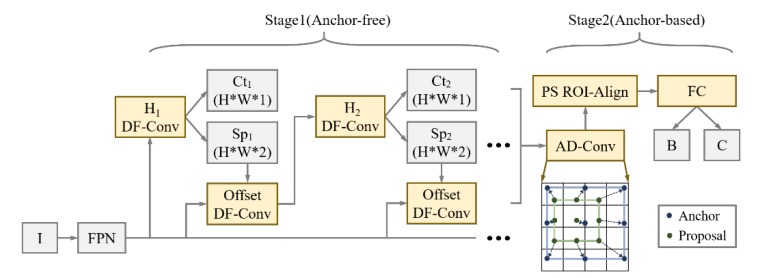
Cascaded Guided-Region Proposal Network (CG-RPN) with semantics-guided refinement.

Region anchors, which are the cornerstone of learning-based detection, play a role in predicting proposals from predefined fixed-size candidates. Selecting positive instances from a large set of densely distributed anchors manually is time-consuming and limited to finite size variance. Some defect instances contained extreme sizes and regression distance between ground truth and anchor may be great. Therefore, in the first stage, the detection pipelines of this paper focus on guided anchor (GA-Net) [32] mechanism to predict centers and sizes of proposals from FPN outputs and, in the second stage, regression and classification are conducted after feature fusion and alignment by position-sensitive (PS) RoI-Align [33], as shown in Figure 6.

#### 3.3.1. Stage for Proposal Generation

All of the proposals would be regressed to bounding boxes of final prediction, thus the quality of generator is crucial. Following the paradigm of GA-Net, RPN comprised of two branches for location and shape prediction, respectively. Given a FPN input F, the location prediction branch Ct (Center-ness) yields a probability map that indicates the Sigmoid scores for centers of the objects by Conv1*1, while the shape prediction branch Sp (Shape) predicts the location-dependent sizes. This branch will lead shapes to the highest coverage with the nearest ground-truth bounding box. Two channels represent two variables height and width but it is necessary to be transformed by Equation (5) for the large range and instability of them.

(5) $$B_{H}^{j}(i)=Conv\left(\frac{w_{1}\cdot S_{L}^{j}\left(B_{L}^{j}(i+1)\right)+w_{2}\cdot S_{H}^{j}\left(B_{H}^{j}(i+1)\right)}{w_{1}+w_{2}+\epsilon }\right)$$

in which,

(6) $$\beta_{w}=\frac{w_{\max}}{w_{\min}}\cdot \prod_{i=1}^{L}s_{i},\;\beta_{h}=\frac{h_{\max}}{h_{\min}}\cdot \prod_{i=1}^{L}s_{i}$$

where (*w*, *h*) are the output of shape prediction, *s_i_* is the stride for different layer *L,* and *β* is a scale factor, depending on size of image data. The nonlinear mapping normalizes the shape space from approximately (0, 2000) to (0, 1), leading to an easier and stable learning target. Since it allows for arbitrary aspect ratios, our scheme can better capture those extremely tall or wide defect instances and encode them to a consistent representation.

**Figure 7:**
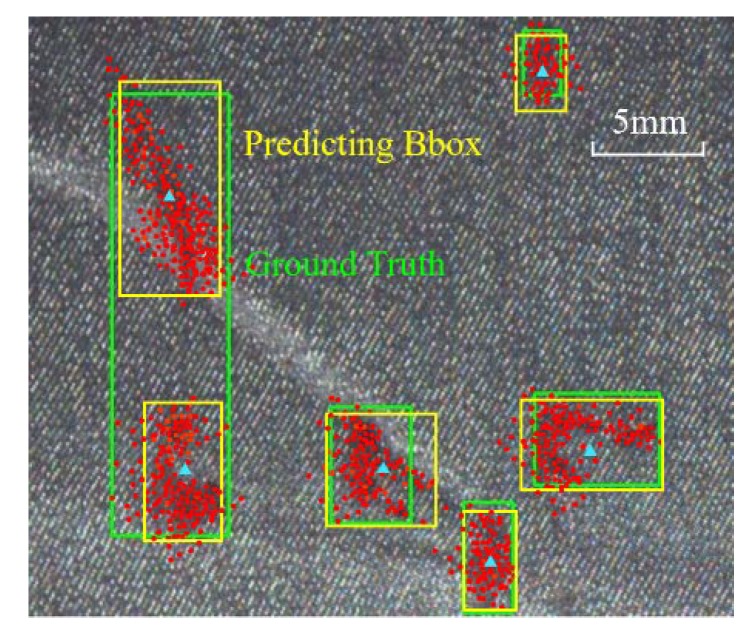
Effective receptive field from deformable extraction in guided localization.

A further size-adaptation offset map is introduced, as the anchor shapes are supposed to be changeable to capture defects within different ranges. With these branches, a set of anchors are generated by selecting the center-ness whose predicted probabilities are above a slightly lower threshold and several shapes with the top probability at each of the chosen feature position. Subsequently, the center-ness threshold is increased for the refinement of the next anchor-selection module and the policy for shapes of anchors is unchanged. Increasing thresholds are set in different sub-modules to include more probable central points and deal with the misalignment of extreme shaped defects. Center-ness is shifted and updated after DF convolution. By this way, with the aid of dense sampler, a large number of anchors are selected, suppressed, and regressed to 256 proposals for stage two. As shown in Figure 7, yellow boxes are the maximum-IOU (Intersection Over Union) candidates chosen after coarsely locating irregularly-shaped defect instances, which are named cascaded guided RPN (CG-RPN). The red points denote strongly semantic feature positions and blue triangles represent centroids of them.

#### 3.3.2. Stage for Bounding Box Generation

**Figure 8:**
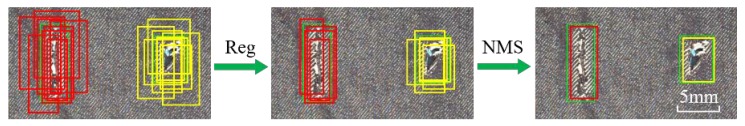
From positive anchors to proposals and finally to bounding boxes.

The bounding boxes need to be regressed and filtered from a large amount of low-quality anchors and Non-maximum Suppression (NMS) [34] are operated to filter the overlaying ones by local maximum search, whose result is shown in Figure 8. On the other hand, multi-classifying branch fixes the output size of full-connection layer so RoI-Align along with adapting pooling aggregates different fields into shape-identical feature map.

Moreover, the loss of the proposed detector is divided into four parts: location branch and final classification branch are similar and Focal Loss (FL) [35] and Cross Entropy (CE) Loss optimize them. Shape branch, which compares IOUs by manually assigning central area ranges, use Bounded IOU Loss (BI) conducted by height and width and regression branch is common with Smooth L1 Loss (SM), as following:

(7) $$L=FC_{loc}+BI_{shape}+CE_{cls}+SM_{reg}$$

where *CE* and *SM* are applied in stage two and smoothing factor is set 0.04 to avoid sensitivity to outliers and suppress gradient explosion. *BI* derived from *SM* along with parameters of bounding boxes. *γ* in *FC* loss is 2 for balancing positive and negative samples.

### 3.4. Evaluation Metrics for Imbalanced Detection for Defects

**Figure 9:**
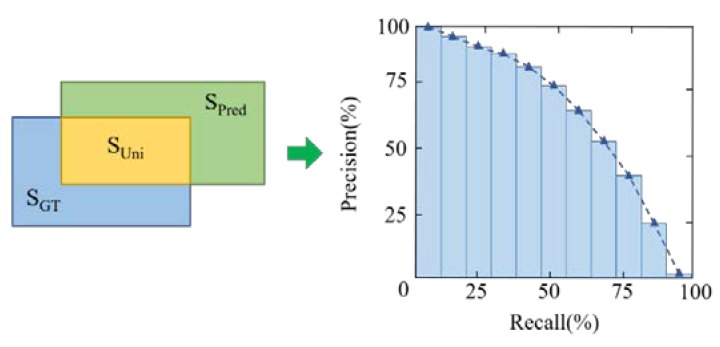
Metrics for performance of imbalanced detection.

Imbalanced detection needs to be evaluated by average recognition precision and variance fluctuation among different categories. Similarity, between ground truth and predicting bounding boxes is proportional to the recognition ability of detectors. Similarity is denoted by IOU based on the Jaccard Index, which evaluates the overlap between two bounding boxes, as shown in Figure 9 and Equation (8), By comparing with confidence threshold, IOU of every instance in every category would divide the prediction results into three aspects: True Positive (TP) denotes a correct detection with IOU ≥ threshold, FP denotes a wrong detection with IOU < threshold and FN reveals a ground truth not detected. After counting the number of instances in distinguished quality, a balanced metrics of AP (Average Precision) could be calculated and used for representing the average performance of detection. In Figure 1, the dashed curve is the Recall-Precision Curve, which is denoted by blue bins and whose area is no more than 1 for facilitating consistency with probability. AP could be calculated in Equation (9), in which b is the number of bins and here is 11. *P* and Δ*r* are the height and width of each bin, respectively.

(8) $$IOU_{\left(c,i\right)}=\frac{\mathrm{area}\left(S_{Pred}\cap S_{GT}\right)}{\mathrm{area}\left(S_{Pred}\cup S_{GT}\right)}$$

(9) $$AP=\sum_{b=1}^{n}P(b)\Delta r(b)$$

For each single class, precision is the ability of a model to only identify the relevant objects. It is the percentage of correct positive predictions and it is given by TP/(TP + FP). Recall is the ability of a model to find all of the relevant cases (all ground truth bounding boxes). It is the percentage of true positive detected among all of the relevant ground truths and it is given by TP/(TP + FN). AP is different among every category due to the imbalance of fabric defects in inter-class and intra-class. Firstly, the mean AP of all categories could be used as the overall performance of detectors and it is named mAP (usually use AP as default). Secondly, for inter-class imbalance, which means that the data distribution of every class significantly differs from each other, the PR curve is better than ROC (Receiver Operating Characteristic), since ROC considers both positive and negative examples. AP focuses on positive ones and Variance Precision (VP) illustrates the inter-class accuracy stability, as in Equation (10). Thirdly, intra-class imbalance mainly lies in size-variance and the AP for small, medium, and large objects is divided by cross scale 96^2^ and 256^2^.

(10) $$VP=\frac{1}{C}\sqrt{\sum_{k=1}^{C}{\left(AP_{k}-mAP\right)}^{2}}$$

## 4. Experiments and Discussion

### 4.1. Experimental Settings

The experiments are performed on FBDF to validate whether the modules above could solve the imbalance of the textile industrial scenes. The validation dataset is evenly split from the whole at splitting ratio of 0.2. Additionally image size does not need to be resized and without changing the aspect ratio. Mini-batch stochastic gradient descent and batch normalization [36] are implemented over two TITAN RTX GPUs with 18 images per worker on one GPU. The training epochs are uniformed to 20 and learning rate is decreased every four epochs with a decreasing rate of 0.1. The evaluation metrics is AP at different IOU thresholds (from 0.5 to 0.95). 200 instances of every class from these images are randomly split as the pre-trained classification dataset, with which all backbones of the architectures are initialized, in order to further strengthen feature extraction.

### 4.2. Main Result

The proposed scheme can be evaluated with other state-of-the-art well-designed methods, as the comparison in Table 4. MC-Net and CI-FPN along with Mixed-16, which is short for Defect-Net and composite interpolating FPN along with mixed convolution network of 16 layers, achieves a remarkable improvement, especially for small defects. It reports a testing AP of 72.6%, an improvement of 12.1% over 60.5%, being achieved by cascaded FPN under the same setting. When using the light-weight backbone (i.e., Mixed-16), the AP improvement over Mixed-16 is 6.7% and AP_S_ improves 29.1%, which prove the availability of mixed convolution. The phenomenon of the increasing from 60.5% (Cascaded R-CNN) to 65.9% (DF-Net + CI-FPN + ResNet50) and 65.9% to 72.6% (MC-Net + CI-FPN + Mixed-16) prove the guessing of over-fitting from large-scale backbone, especially for small defects. The VP of generic baselines is larger than that of the proposed architecture on average. VP of MC-Net along with CI-FPN and Mixed-16 is the lowest and FCOS is the second one, which reveals that the range of AP for different categories is narrow and distribution is relatively even. However, it does not mean that the more VP is, the higher accuracy detector possesses. Take YOLOv3 as an example, the experiments show that the APs of 11 classes are less than 30% in spite of 10.8 in VP. Additionally, Cascaded-FPN gains 0.3 VP larger than Libra-FPN-RetinaNet, but 4.5% AP larger than that. Therefore, the ability for addressing the imbalance of detectors should be evaluated by a combination of AP and VP.

### 4.3. Ablation Experiments

**Figure 10:**
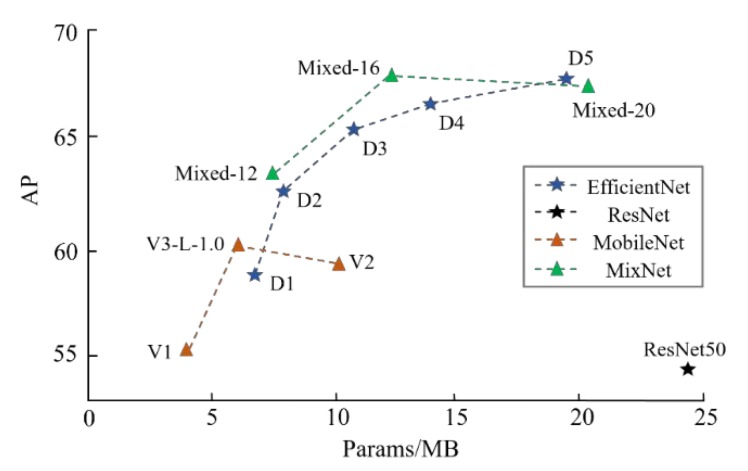
Model sizes with Average Precision (AP) for FBDF of different high-efficiency backbones upon Faster R-CNN and FPN.

Backbone extraction. As MC-Net uses a light-weight powerful backbone, Figure 10 reveals how much each of them contributes to the accuracy and efficiency improvements. Faster R-CNN along with FPN is our baseline for comparison of different backbone. First, the RestNet series are heavy and low-efficiency, which achieve a relatively low accuracy and ResNet-101, along with ResNet-152 are even worse, and are thus are not shown in the figure. When replacing with MobileNet, AP increases from 53% to 61% over MobileNetv3-Large [44] without cropping the images. Mixed series achieve a similar performance with EfficientNet series and Mixed-16 gains the top AP of 67.2% based on FBDF and slightly decreasing from Mixed-20 is due to the redundancy of the weights.

Along with the variant improvement for Faster R-CNN, the MC module is still efficient for other detectors in defect detection. In Table 5, Cascaded FPN gains 6.0% promotion from 66.5% on the ResNet-50 backbone and the average improvement of small instances is 5.5%, which proves MC-Net could extract more and deep feature from the low-salience defects.

**Figure 11:**
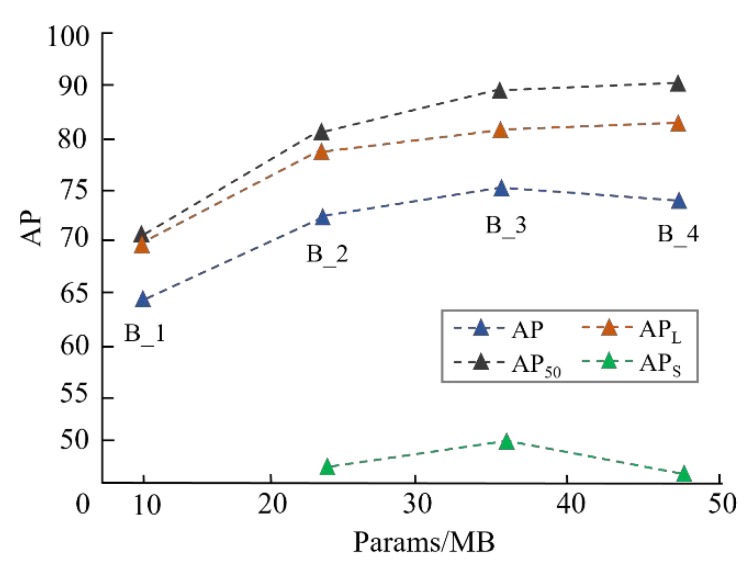
The performance of different configurations of CI-FPN with CG-RPN modules.

Neck fusion. In Figure 11, the AP of composite interpolating FPN is rising with the model complexity expanding. B_n_ is short for n blocks of two-way information flow. Notably, when three blocks are employed along with inter-layer and intra-block cross-scale connections, the scheme is the most accurate one, with 72.3% (AP), 50.9% (AP_S_), and 36.4 MB training parameters.

Proposals generation. With the deployment of CG-RPN, three feature adaption modules would refine the anchor centers and shapes in stage one. The center-ness thresholds are 0.3, 0.5, and 0.7 in different cycles and, in regression branch, every position in feature map choose three anchors with aspect ratios of 0.5, 1.0, and 2.0 to enlarge the search space. In Figure 11, the left one is from common Faster R-CNN and the right one is from CG-RPN and less low-quality proposals is reserved here. In Table 6, different center-ness configurations are displayed and the tuple (0.3, 0.5, 0.7) is better than the others in AP, since it introduces more computing parameters and relaxes the hard border of whether belonging to positive instances.

**Figure 12:**
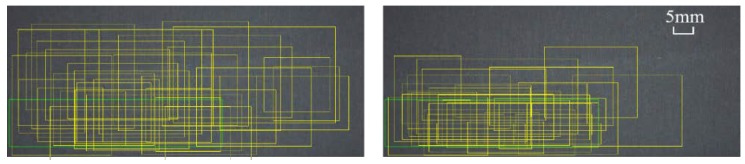
Sample RPN proposals from Faster R-CNN and C-G RPN.

**Figure 13:**
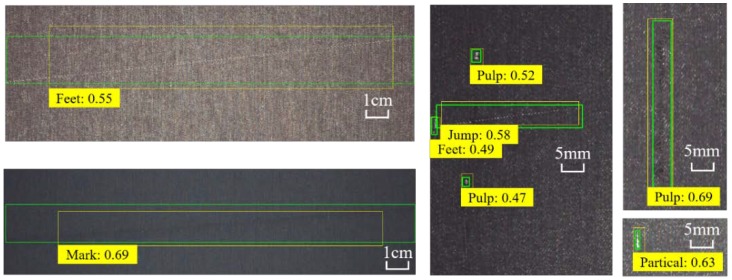
Visualization of the best bounding boxes of defects from MC-Net along with CI-FPN.

Additionally, from the pre-trained model of MC-Net with CI-FPN, several bounding boxes and confidence values are shown in Figure 12 and Figure 13.

For Area Under Curve (AUC), the MC-Net along with Mixed-16 gains better performance than AP: 76.9% for mean AUC, 55.4% for small defects and 82.6% for large defects. For ConerNet and CenterNet, the mean AUC increases 3.5% and 4.3% and some small promotions in accuracy appear in other detecting systems. However, in textile industry, positive examples draw more attention than negative examples and a detector that is robust to sensitive metrics. When negative examples increase a lot, the curve does not change a lot, which is equivalent to generating a large number of FP. In the context of imbalanced categories, the large number of negative cases makes the growth of FPR (FPR = FP/(FP + TN); TPR = TP/(TP + FN)) not obvious, resulting in an ROC curve that shows an overly optimistic effect estimate. Finally, misdetection would lead to constant interruptions of machine tools, which results in low efficiency in manufacturing. Therefore, in this work, the ROC curve is replaced for the PR curve.

## 5. Conclusions

This study solves the problem of the imbalanced detection for fabric defect. Firstly, a large-scale, publicly available dataset for defect detection in optical fabric defect images is released, which enables the community to validate and develop data-driven defect detection methods. Secondly, several modules to refine traditional inefficient network are designed, including mix convolutional backbone, interpolating FPN, and cascaded guided anchor, etc., in order to improve recognition performance of occluded and size-variant defects Finally, the study shows the importance of these frameworks in defect detecting and provides a scheme for precisely meeting the needs of the textile industry.

## Figures and Tables

**Table 1:** Samples of different classes of Fabric Defects (FBDF).

Feet	Particles	Knots	Spandex	Rg-Warp	Stains
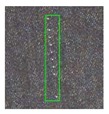	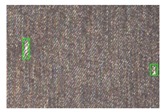	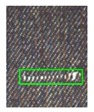	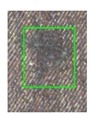	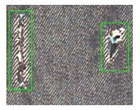	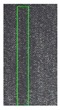

**Table 2:** Specification for mixed convolution (MC) backbone.

Input	Operator	EXP Size	AF	SE	FPN
2446 × 1000 × 3	3 × 3	-	RE	-	-
896 × 448 × 16	3 × 3	{40, 72}	RE	-	-
448 × 224 × 24	3 × 3, 5 × 5	{72, 72}	HS	√	√
224 × 112 × 40	3 × 3	{72, 120, 240}	HS	√	-
112 × 56 × 80	3 × 3, 5 × 5, 7 × 7	{200, 240}	HS	√	√
56 × 28 × 112	3 × 3, 5 × 5, 7 × 7, 9 × 9, 11 × 11	{240, 480}	RE	√	√
28 × 14 × 160	3 × 3, 5 × 5	{480, 672}	HS	√	√

**Table 3:** Performance of the state-of-the-art FPN baselines on FBDF.

Baseline	Backbone	AP	AP_50_	AP_75_
FPN	ResNet-50	34.29	52.01	36.68
NAS-FPN	AmoebaNet	36.16	55.74	40.77
PANet	VGG-16	39.51	60.17	42.14
EfficientDet	EfficientNet-B4	46.39	65.68	50.27
CBNet	ResNet-50	44.22	60.38	46.96

**Table 4:** Accuracy of different detectors on FBDF testing set.

Method	Backbone	AP	AP_50_	AP_75_	AP_S_	AP_L_	VP
Faster R-CNN	VGG-16	42.6	57.7	45.8	22.4	53.6	14.2
Faster + FPN	ResNet-50	53.3	69.0	57.7	39.3	64.7	13.8
RetinaNet + FPN	ResNet-50	55.7	73.3	60.9	42.7	66.5	12.6
YOLOv3 [37]	DarkNet-53	35.5	52.5	36.2	19.4	50.5	10.8
SSD-513 [38]	ResNet-101	32.9	54.9	34.1	12.2	48.4	12.9
Cascaded [39] + FPN	ResNet-50	60.5	75.2	66.3	47.1	71.0	11.5
CornerNet [40]	Hourglass-52	36.4	53.0	39.8	19.9	51.2	13.0
CenterNet [41]	Hourglass-52	39.5	57.5	40.6	22.7	54.3	12.6
Libra-FPN-RetinaNet [42]	ResNet-50	56.9	71.4	60.2	38.5	68.9	11.2
FCOS [43]	ResNet-50	33.0	49.8	34.4	19.2	44.3	10.3
**MC-Net + CI-FPN**	**ResNet-50**	**65.9**	**79.5**	**68.0**	**48.3**	**77.3**	**10.7**
**MC-Net + CI-FPN**	**Mixed-16**	**72.6**	**86.3**	**73.6**	**50.9**	**80.4**	**9.7**

**Table 5:** Performance of Mixed-16 applied in some generic detectors.

Method	Backbone	AP	AP_50_	AP_75_	AP_S_	AP_L_
Faster + FPN	Mixed-16	59.2(5.9)	74.2	63.6	43.0	70.1
RetinaNet + FPN	Mixed-16	61.9(6.2)	79.8	66.7	46.5	73.0
Cascaded R-CNN	Mixed-16	63.6(4.5)	78.4	80.1	47.5	72.8
Cascaded + FPN	Mixed-16	66.5(6.0)	83.5	72.5	59.8	77.9

**Table 6:** Performance of CG-RPN with different center-ness threshold tuples.

Tuple	Mean IOU (Post)	AP	AP_S_
(0.5)	46.5	71.4	39.1
(0.5, 0.7)	**59.7**	**75.8**	**44.8**
(0.3, 0.5)	53.6	72.9	41.3
(0.3, 0.5, 0.7)	**56.8**	**76.1**	**46.5**

## References

[1] Ahmed J., Gao B., Woo W.L. (2019). Wavelet Integrated Alternating Sparse Dictionary Matrix Decomposition in Thermal Imaging CFRP Defect Detection. IEEE Trans. Ind. Inform..

[2] Gao B., Lu P., Woo W.L., Tian G.Y., Zhu Y., Johnston M. (2019). Variational Bayesian Sub-group Adaptive Sparse Component Extraction for Diagnostic Imaging System. IEEE Trans. Ind. Electron..

[3] Wang Y., Gao B., lok Woo W., Tian G., Maldague X., Zheng L., Guo Z., Zhu Y. (2018). Thermal Pattern Contrast Diagnostic of Microcracks With Induction Thermography for Aircraft Braking Components. IEEE Trans. Ind. Inform..

[4] Hamdi A.A., Sayed M.S., Fouad M.M., Hadhoud M.M. Unsupervised patterned fabric defect detection using texture filtering and K-means clustering. Proceedings of the International Conference on Innovative Trends in Computer Engineering.

[5] Mei S., Wang Y., Wen G.J. (2018). Automatic Fabric Defect Detection with a Multi-Scale Convolutional Denoising Autoencoder Network Model. Sensors.

[6] Seker A., Peker K.A., Yuksek A.G., Delibaş E. Fabric defect detection using deep learning. Proceedings of the 24th Signal Processing and Communication Application Conference (SIU).

[7] Li Z., Peng C., Yu G., Zhang X., Deng Y., Sun J. (2018). Detnet: A backbone network for object detection. arXiv.

[8] Ren S., He K., Girshick R., Sun J. (2017). Faster R-CNN: Towards Real-Time Object Detection with Region Proposal Networks. IEEE Trans. Pattern Anal. Mach. Intell..

[9] Liu Y., Li H., Yan J., Wei F., Wang X., Tang X. Recurrent Scale Approximation for Object Detection in CNN. Proceedings of the IEEE International Conference on Computer Vision (ICCV).

[10] Singh B., Davis L.S. An analysis of scale invariance in object detection snip. Proceedings of the IEEE Conference on Computer Vision and Pattern Recognition.

[11] Girshick R. Fast R-CNN. Proceedings of the 2015 IEEE International Conference on Computer Vision (ICCV).

[12] He K., Gkioxari G., Dollár P., Girshick R. Mask R-CNN. Proceedings of the IEEE International Conference on Computer Vision.

[13] Jiang B., Luo R., Mao J., Xiao T., Jiang Y. Acquisition of localization confidence for accurate object detection. Proceedings of the European Conference on Computer Vision (ECCV).

[14] Li K., Wan G., Cheng G., Meng L., Han J. (2018). Object detection in optical remote sensing images: A survey and a new benchmark. ISPRS J. Photogramm. Remote Sens..

[15] Lin T.Y., Maire M., Belongie S., Hays J., Perona P., Ramanan D., Zitnick C.L. Microsoft coco: Common objects in context. Proceedings of the European Conference on Computer Vision.

[16] Everingham M., Van Gool L., Williams C.K., Winn J., Zisserman A. (2010). The pascal visual object classes (voc) challenge. Int. J. Comput. Vis..

[17] Tan M., Le Q.V. (2019). Mixconv: Mixed depthwise convolutional kernels. arXiv.

[18] Hu J., Shen L., Sun G. Squeeze-and-excitation networks. Proceedings of the IEEE Conference on Computer Vision and Pattern Recognition.

[19] Chen Y., Dai X., Liu M., Chen D., Yuan L., Liu Z. (2019). Dynamic Convolution: Attention over Convolution Kernels. arXiv.

[20] He K., Zhang X., Ren S., Sun J. Deep residual learning for image recognition. Proceedings of the IEEE Conference on Computer Vision and Pattern Recognition.

[21] Howard A.G., Zhu M., Chen B., Kalenichenko D., Wang W., Weyand T., Adam H. (2017). Mobilenets: Efficient convolutional neural networks for mobile vision applications. arXiv.

[22] Zhang X., Zhou X., Lin M., Sun J. Shufflenet: An extremely efficient convolutional neural network for mobile devices. Proceedings of the IEEE Conference on Computer Vision and Pattern Recognition.

[23] Sandler M., Howard A., Zhu M., Zhmoginov A., Chen L.C. Mobilenetv2: Inverted residuals and linear bottlenecks. Proceedings of the IEEE Conference on Computer Vision and Pattern Recognition.

[24] Borji A., Cheng M.M., Jiang H., Li J. (2015). Salient object detection: A benchmark. IEEE Trans. Image Process..

[25] Borji A., Itti L. (2012). State-of-the-art in visual attention modeling. IEEE Trans. Pattern Anal. Mach. Intell..

[26] Lin T.Y., Dollár P., Girshick R., He K., Hariharan B., Belongie S. Feature pyramid networks for object detection. Proceedings of the IEEE Conference on Computer Vision and Pattern Recognition.

[27] Liu S., Qi L., Qin H., Shi J., Jia J. Path aggregation network for instance segmentation. Proceedings of the IEEE Conference on Computer Vision and Pattern Recognition.

[28] Liu Y., Wang Y., Wang S., Liang T., Zhao Q., Tang Z., Ling H. (2019). CBNet: A Novel Composite Backbone Network Architecture for Object Detection. arXiv.

[29] Ghiasi G., Lin T.Y., Le Q.V. NAS-FPN: Learning scalable feature pyramid architecture for object detection. Proceedings of the IEEE Conference on Computer Vision and Pattern Recognition.

[30] Zoph B., Vasudevan V., Shlens J., Le Q.V. Learning transferable architectures for scalable image recognition. Proceedings of the IEEE Conference on Computer Vision and Pattern Recognition.

[31] Tan M., Pang R., Le Q.V. (2019). Efficientdet: Scalable and efficient object detection. arXiv.

[32] Wang J., Chen K., Yang S., Loy C.C., Lin D. Region proposal by guided anchoring. Proceedings of the IEEE Conference on Computer Vision and Pattern Recognition.

[33] Zhu Y., Zhao C., Wang J., Zhao X., Wu Y., Lu H. CoupleNet: Coupling global structure with local parts for object detection. Proceedings of the IEEE International Conference on Computer Vision.

[34] Neubeck A., Van Gool L. Efficient non-maximum suppression. Proceedings of the 18th International Conference on Pattern Recognition (ICPR’06).

[35] Lin T.Y., Goyal P., Girshick R., He K., Dollár P. Focal loss for dense object detection. Proceedings of the IEEE International Conference on Computer Vision.

[36] Ioffe S., Szegedy C. (2015). Batch normalization: Accelerating deep network training by reducing internal covariate shift. arXiv.

[37] Redmon J., Farhadi A. (2018). YOLOv3: An incremental improvement. arXiv.

[38] Liu W., Anguelov D., Erhan D., Szegedy C., Reed S., Fu C.Y., Berg A.C. SSD: Single shot multibox detector. Proceedings of the European Conference on Computer Vision.

[39] Cai Z., Vasconcelos N. Cascade R-CNN: Delving into high quality object detection. Proceedings of the IEEE Conference on Computer Vision and Pattern Recognition.

[40] Law H., Deng J. Cornernet: Detecting objects as paired keypoints. Proceedings of the European Conference on Computer Vision (ECCV).

[41] Duan K., Bai S., Xie L., Qi H., Huang Q., Tian Q. Centernet: Keypoint triplets for object detection. Proceedings of the IEEE International Conference on Computer Vision.

[42] Pang J., Chen K., Shi J., Feng H., Ouyang W., Lin D. Libra R-CNN: Towards balanced learning for object detection. Proceedings of the IEEE Conference on Computer Vision and Pattern Recognition.

[43] Tian Z., Shen C., Chen H., He T. (2019). FCOS: Fully Convolutional One-Stage Object Detection. arXiv.

[44] Howard A., Sandler M., Chu G., Chen L.C., Chen B., Tan M., Le Q.V. (2019). Searching for mobilenetv3. arXiv.

